# Oxygen-Scavenging Multilayered Biopapers Containing Palladium Nanoparticles Obtained by the Electrospinning Coating Technique

**DOI:** 10.3390/nano9020262

**Published:** 2019-02-14

**Authors:** Adriane Cherpinski, Piotr K. Szewczyk, Adam Gruszczyński, Urszula Stachewicz, Jose M. Lagaron

**Affiliations:** 1 Novel Materials and Nanotechnology Group, Institute of Agrochemistry and Food Technology (IATA), Spanish Council for Scientific Research (CSIC), Calle Catedrático Agustín Escardino Benlloch 7, 46980 Paterna, Spain; adricherpinski@iata.csic.es; 2 AGH University of Science and Technology, International Centre of Electron Microscopy for Materials Science, Faculty of Metals Engineering and Industrial Computer Science, Al. A. Mickiewicza 30, 30-059 Kraków, Poland; pszew@agh.edu.pl (P.K.S.); gruszcz@agh.edu.pl (A.G.); ustachew@agh.edu.pl (U.S.)

**Keywords:** polyhydroxyalkanoates, polycaprolactone, biopapers, palladium nanoparticles, oxygen scavengers, electrospinning, fiber based packaging

## Abstract

The main goal of this study was to obtain, for the first time, highly efficient water barrier and oxygen-scavenging multilayered electrospun biopaper coatings of biodegradable polymers over conventional cellulose paper, using the electrospinning coating technique. In order to do so, poly(3-hydroxybutyrate) (PHB) and polycaprolactone (PCL) polymer-containing palladium nanoparticles (PdNPs) were electrospun over paper, and the morphology, thermal properties, water vapor barrier, and oxygen absorption properties of nanocomposites and multilayers were investigated. In order to reduce the porosity, and to enhance the barrier properties and interlayer adhesion, the biopapers were annealed after electrospinning. A previous study showed that electrospun PHB-containing PdNP did show significant oxygen scavenging capacity, but this was strongly reduced after annealing, a process that is necessary to form a continuous film with the water barrier. The results in the current work indicate that the PdNP were better dispersed and distributed in the PCL matrix, as suggested by focus ion beam-scanning electron microscopy (FIB-SEM) experiments, and that the Pd enhanced, to some extent, the onset of PCL degradation. More importantly, the PCL/PdNP nanobiopaper exhibited much higher oxygen scavenging capacity than the homologous PHB/PdNP, due to most likely, the higher oxygen permeability of the PCL polymer and the somewhat higher dispersion of the Pd. The passive and active multilayered biopapers developed here may be of significant relevance to put forward the next generation of fully biodegradable barrier papers of interest in, for instance, food packaging.

## 1. Introduction

Active technologies, when applied to packaging refer to the incorporation of certain additives into the packaging structure. These additives may be loose as sachets within the design, attached to the inside part or, more recently, dispersed as an additive within the packaging materials, in order to maintain or even extend product quality and shelf-life [1].

Permeated or head space oxygen in packaged foods, beverages, and pharmaceuticals can promote a range of oxidative degradation reactions and support microbial growth, ultimately impacting on product quality and shelf-life. Oxygen-scavenging active packaging systems have therefore been explored, to control headspace oxygen content [2].

The application of oxygen scavengers is one of the most important active packaging technologies, which aim to remove any residual oxygen that is present in the food packaging. In some cases, the residual levels of oxygen in the package can be reduced to < 0.01 vol %, and actively controlled, which is not possible with other packaging systems [3].

Oxygen scavengers are by far the most commercially important sub-categories of active packaging, and the market has been growing steadily over the last few years. Almost all oxygen scavenger sachets used commercially are based on the principle of iron oxidation. On the other hand, oxygen-scavenging film is a more promising emerging packaging technology, because it contains the active material within the film, and consumers are not in favor of having foreign objects such as sachets in the lining of their product packaging. With oxygen scavenger films, the consumer cannot physically see the oxygen scavenger materials, yet are able to experience its benefits [4,5,6].

The incorporation of scavengers into packaging films is a better way of resolving sachet-related problems. Scavengers may either be imbedded into a solid, dispersed in the plastic, or introduced into various layers of the package, including adhesive, lacquer, or enamel layers. Multi-layer oxygen scavengers more effectively absorb oxygen than single-layer scavenging systems [7].

Several new oxygen-scavenging technologies have been developed over the last decade, incorporating active substances and metals directly into packaging films or containers [8]. However, only a few of them have been successfully implemented in real food systems, due to, for instance, in the case of metals that function by chemical reduction, low reaction capacities and the need for triggering mechanisms, among other factors. Consequently, real application studies demonstrating the benefits of alternative oxygen-scavenging systems to particular food products are rather rare [9].

Recently, Hutter et al. [10] developed an oxygen scavenging film based on a catalytic system with palladium (CSP), which is able to reduce residual headspace oxygen very quickly. Palladium, in very low dosages, catalyzes the oxidation of hydrogen into water, and thus can remove the residual oxygen in the headspace of a modified atmosphere package containing hydrogen. Catalytic systems based on palladium have also been reported to have other interesting applications, such as the construction of complex molecules [11,12,13,14].

The main difficulties of this approach are the dispersion of the scavenger in the matrix, the accessibility of the scavenger to oxygen, and the necessity of an activation system for the oxygen absorption reaction. Without an activation system, the oxygen-scavenging capacity of the active film would be consumed during storage, before the packaging is used [15].

In addition, consumer trends for better quality, fresh-like, and convenient food products have intensified over the last decade. Therefore, a variety of active packaging technologies have been developed to provide better quality, wholesome, and safe foods, and also to limit package-related environmental pollution and disposal problems.

Recently, the environmental impact of persistent plastic packaging wastes is raising general global concern, since disposal methods are limited. Biopolymers have been considered as a potential environmentally-friendly substitute for the use of non-biodegradable and non-renewable plastic packaging materials [16]. 

Polycaprolactone (PCL) is petroleum-based, but it can be degraded by microorganisms, and the polyhydroxyalkanoate (PHA) homopolymer called poly(hydroxybutyrate) (PHB) is produced from biomass or renewable resources, and it is readily biodegradable [17]. The aim of this emerging and developing field is to change the nature of polymer products and to minimize the environmental impact. Various approaches are currently being investigated for possible polymers that may be utilized to design adequate environmentally friendly packaging [18].

Electrospinning is a feasible, efficient, and convenient technique for obtaining biopolymer-active nanofibers of interest in many application fields, such as active packaging, and since recently, it has also been scaled up for mass production [19,20,21,22,23]. Many factors influence fiber morphology and diameter, including solid concentrations, types of solvent, surface tension, additivation, solution viscosity, polymer molecular weight, flow rate, injector design, spinneret diameter, solution conductivity, injector to collector distance, and applied voltage. Of the many parameters discussed, concentration/solution viscosity, surface tension, and conductivity are probably the most important factors affecting the final fiber morphology and diameter [24,25,26]. A previous study [15] dealt with the development of a monolayer of oxygen-scavenging electrospun PHB containing palladium nanoparticles (PdNP). This monolayer demonstrated oxygen scavenging, but after annealing of the fibers to reduce porosity and to generate a water barrier, the material reduced the oxygen scavenging capacity to a significant extent.

The present work focuses, for the first time, on the preparation of significantly enhanced oxygen-scavenging bilayered coatings of PHB and PCL electrospun fibers, so-called biopapers, containing PdNPs, so-called nanobiopapers, deposited on a cellulose paper, to derive an optimized passive and active coating of interest in biodegradable fiber-based packaging.

## 2. Materials and Methods 

### 2.1. Materials

The microbial homobiopolyester PHB, P226F grade, was obtained from Biomer (Krailling Germany). This grade is certified as both compostable and food contact. It has a density of 1.25 g/cm^3^ and a melt flow rate (MFR) of 10 g/10 min when tested at 180 °C using a 5 kg load. 

Polycaprolactone (PCL) (Mw: Mn 80 kDa), 2,2,2-trifluoroethanol (TFE) with 99% purity and D-limonene with 98% purity were purchased from Sigma-Aldrich S.A. (Madrid, Spain). Hexadecyltrimethylammonium bromide (CTAB) with 99% purity and palladium (Pd) nano-powder, <25 nm particle size measured by transmission electronic microscopy (TEM) and ≥99.5% trace metals basis, were also purchased from Sigma-Aldrich S.A. Chloroform (≥99%) and 1-butanol (99.5%) were purchased from AppliChem. All products were used as received without further purification. CTAB was selected as surfactant for PHB, because it is currently permitted for food contact applications by FDA and EFSA.

The conventional cellulose fiber-based packaging substrate was prepared using commercial bleached Kraft eucalyptus pulp as raw material, which was kindly provided by Ence-Celulosas y Energia S.A. (Madrid, Spain). Briefly, the pulp was disintegrated in a pulp disintegrator for 1 h at 3000 rpm to achieve a consistency of 1.5%. Paper sheets of 700 × 16 mm^2^ with a final grammage of 75 g/m^2^ were fabricated in an isotropic Rapid-Kӧthen sheet former and conditioned at 23 °C and 50% of relative humidity (RH) according to ISO standard 187. The grammage and thickness were evaluated following ISO standards 536 and 534, respectively. Further details can be found in previous research [22,27].

### 2.2. Preparation of the Films

Before electrospinning, the PHB solution was prepared by dissolving 10 wt % in TFE under magnetic stirring conditions at 50 °C. The PHB/PdNP suspension was prepared by adding CTAB (0.25 wt. % in the fibers) surfactant and PdNP (1 wt. % in the fibers) to the PHB solution.

PCL was prepared by dissolving 10 wt % in butanol:chloroform (25:75) under magnetic stirring conditions at room temperature. To prepare the solution of PCL/PdNP, PdNP were added (1 wt % in the fibers) in the previous solution and dissolved while magnetically stirring.

The electrospinning device used was a high throughput Fluidnatek^®^ LE-500, used in lab mode with temperature and relative humidity control pilot plant equipment from Bioinicia S.L. (Valencia, Spain), a variable 0–60 kV dual polarizer high-voltage power supply, and a scanning injector, to obtain a homogeneous deposition of fibers. To obtain the electrospun PHB layers, the biopolymer solution was transferred to a 30 mL plastic syringe and coupled by a Teflon tube to a stainless-steel needle (∅ = 0.9 mm) that was connected to the power supply. PHB and PCL solutions were electrospun at 25 °C and 30% RH on a flat metallic collector, for 2 and 1 h under a steady flow-rate of 6 mL/h and 2 mL/h, respectively, using a motorized injector, scanning vertically toward a metallic grid collector. A distance between the injector and collector was both optimal at 15 cm, and the applied voltage was 16 kV and 12 kV.

The electrospun PHB and PCL coatings were subjected to an annealing post-processing step below the polymers’ melting points, at temperatures of 160 °C and 50 °C, respectively using a hydraulic press 4122-model from Carver, Inc. (Indiana, IN, USA). This post-processing thermal treatment was applied for 5 s without pressure, to ensure the coalescence of the fibers mat into a continuous film. The conditions were selected based on the research conducted in previous works [21,28,29]. 

### 2.3. Characterization of the Films

#### 2.3.1. Film Thickness

Prior to characterization, the whole thickness of all of the structures was measured by using a digital micrometer (Series S00014, having ± 0.001 mm accuracy, from Mitutoyo Corporation (Kawasaki, Japan)). Measurements were performed at three random positions, and values were averaged. All samples were stored before evaluation, in desiccators containing dried silica gel at 25 °C.

#### 2.3.2. Focus Ion Beam Scanning Electron Microscopy (FIB-SEM)

Electrospun samples were prepared for microscopy evaluation by fixing nanofibers deposited on aluminum foil with carbon tape, and they were gold coated with a 5 nm layer, using a rotary-pump sputter coater (Q150RS, Quorum Technologies, UK). The samples were imaged with a scanning electron microscope (SEM), using an accelerating voltage of 3 kV, 0.15 nA current and a working distance of 5 mm. 3D tomography of nanofibers with nanoparticles was achieved by using a dual beam system (NEON CrossBeam 40EsB, Zeiss, Germany) integrating a SEM with a focused ion beam (FIB). The sample stage was tilted at 54° so that the sample surface was perpendicular to the FIB direction [30], as demonstrated in previous research [31]. The 12–14 nm thick cross-sectional slices were milled by using FIB from the nanofiber sample at 30 kV and a beam current of 0.5 nA. [32,33]. The collected SEM images during FIB sectioning were filtered and reconstructed in 3D using Avizo Fire (version 6.3—FEI Edition, U.S.A.). To obtain the 3D reconstructions of the PCL fibers, 50 images and 108 images, respectively, for fiber 1 and 2 were used, maintaining the voxel size (5 × 5 × 14 nm). In the case of the PHB fibers, 50 images and a voxel size of 4 × 4 × 12 nm were used. Additionally, Avizo Fire was used to calculate the Pd nanoparticle concentration in the investigated pieces of electrospun fibers from the 3D reconstructions already obtained. 

#### 2.3.3. Scanning Electron Microscopy

An S-4800 SEM microscope from Hitachi (Tokyo, Japan) was further used to observe the morphology of the electrospun PHB films, and their cross-sections and surfaces. Cross-sections of the samples were prepared by cryo-fracture of the electrospun PHB films in liquid nitrogen. Then, they were fixed to beveled holders by using conductive double-sided adhesive tape, sputtered with a mixture of gold-palladium under a vacuum, and observed using an accelerating voltage of 5 kV.

#### 2.3.4. Transmission Electronic Microscopy

The morphology and distribution of Pd nanoparticles were studied in electrospun fibers directly deposited onto clamping holders, and in the case of the films, on ultrathin microtomed sections as described in reference [15], using a Jeol 1010 (Hitachi, Tokyo, Japan) transmission electronic microscope, at an accelerating voltage of 80 kV. 

#### 2.3.5. Differential Scanning Calorimetry (DSC)

Thermal properties of neat PCL and Pd containing PCL electrospun fibers and films were evaluated by differential scanning calorimetry (DSC) using a Perkin-Elmer DSC 8000 (Waltham, MA, USA) thermal analysis system under a nitrogen atmosphere. The analysis was carried out on ~3 mg of each sample at a heating rate of 10 °C/min, from −25 °C to 125 °C, with subsequent cooling to −25°C. The DSC equipment was calibrated with indium as a standard, and the slope of the thermograms was corrected by subtracting similar scans of an empty pan. Tests were done at least in triplicate.

#### 2.3.6. Thermogravimetric Analysis (TGA)

The TGA was performed in a TG-STDA Mettler Toledo model TGA/STDA851e/LF/1600 analyzer. The samples with an initial weight of typically about 15 mg were heated from 50 to 1300 °C at a heating rate of 10 °C/min under nitrogen/air flow.

#### 2.3.7. Infrared Spectroscopy

Fourier transform infrared spectroscopy (FTIR) spectra were collected coupling the attenuated total reflection (ATR) accessory Golden Gate of Specac, Ltd. (Orpington, UK) to Bruker Tensor 37 FTIR equipment (Rheinstetten, Germany). Single spectra were collected in the wavelength range from 4000 to 600 cm^−1^ by averaging 20 scans at a resolution of 4 cm^−1^.

#### 2.3.8. Water Vapor Permeance

The water vapor permeance was determined by using the ASTM 2011 gravimetric method. To this end, 5 mL of distilled water was placed inside a Payne permeability cup (Ø = 3.5 cm) from Elcometer Sprl (Hermalle-sous-Argenteau, Belgium). The films were placed in the cups so that on one side, they were exposed to 100% RH on the coated side, avoiding direct contact with water. The cups containing the films were then secured with silicon rings and stored in a desiccator at 0% RH using dried silica gel, at 25 °C. Identical cups with aluminum films were used as control samples to estimate water loss through the sealing. The cups were weighed periodically using an analytical balance of ±0.0001 g accuracy. The water vapor transmission rate (WVTR), also called water vapor permeance when corrected for permeant partial pressure, was determined from the steady-state permeation slope obtained from the regression analysis of weight loss data per unit area versus time, in which the weight loss was calculated as the total cell loss minus the loss through the sealing. Measurements were performed in triplicate.

#### 2.3.9. Measurement of Oxygen Scavenging Activity

Round-bottom flasks (to Schlenk) from VidraFoc S.A. (Barcelona, Spain) with a polytetrafluoroethylene (PTFE) stopcock and a headspace volume of 50 cm^3^ was used for the oxygen scavenging measurements. The flasks contained a valve for flushing gas in, and an O_2_-sensitive sensor spot (PSt3, detection limit 15 ppb, 0–100% oxygen) from PreSens (Regensburg, Germany) was glued onto the inner side of the flasks for the oxygen depletion measurements. Electrospun fibers and multilayers containing electrospun fibers with same sample areas were cut (5 × 5 cm^2^) and placed into the flasks. The flask was subsequently flushed for 30 s at 1 bar with a gas mixture containing 1 vol % oxygen, 4 vol % hydrogen, and 95 vol % nitrogen, which was provided by Abelló Linde, S.A. (Barcelona, Spain). The oxygen concentration in the cell was monitored by a non-destructive measurement method, using the OXY-4 mini (PreSens) multi-channel fiber optic oxygen meter for simultaneous read-outs of up to four oxygen sensors, and used with sensors based on a 2 mm optical fiber. Oxygen concentrations over time were measured by linking the light-emitting (600–660 nm) optical fibers to the flasks’ inner sensing spots. The sensor emits a certain amount of luminescence, depending on the oxygen concentration in the cell that is calibrated to yield the concentration by the equipment. All measurements were carried out at 23 °C and 50% RH, simulating typical ambient conditions. 

### 2.4. Statistical Analysis 

The test data were evaluated through analysis of variance (ANOVA) using STATGRAPHICS Centurion XVI v 16.1.03 from StatPoint Technologies, Inc. (Warrenton, VA, USA). Fisher’s least significant difference (LSD) was used at the 95% confidence level (*p* < 0.05). Mean values and standard deviations were also calculated. 

## 3. Results and Discussion

### 3.1. Morphology of the Electrospun PCL Fibers and Films

**Figure 1:**
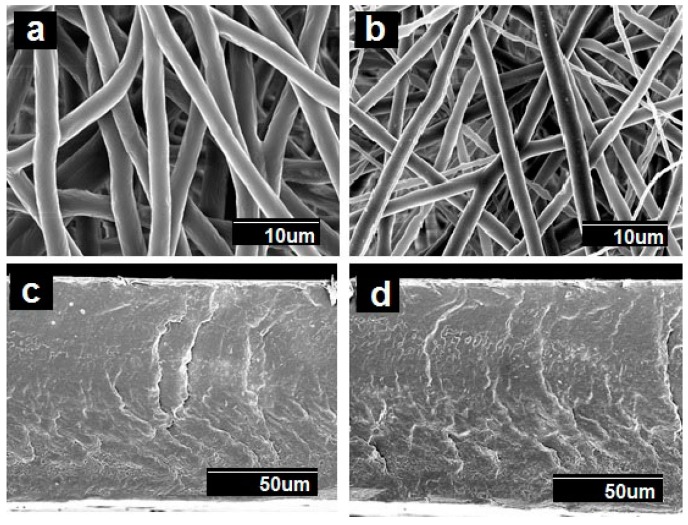
Scanning electron microscopy (SEM) images of the surface view and the cross-section of the polycaprolactone (PCL) fibers, with and without palladium nanoparticles (PdNPs), and their respective annealed films: (**a**) Surface view of the neat PCL fibers; (**b**) Surface view of the PCL/PdNP fibers; (**c**) Cross-section of the neat PCL film; (**d**) Cross-section of the PCL/PdNP film.

As it can be seen from the observation of Figure 1a,b, a narrow distribution of fiber diameter with an average at 2.75 ± 0.4 μm in PCL fibers, and 2.25 ± 0.7 μm in PCL/PdNP fibers was observed. The surface of the formed fibers was seen to be smooth and without beaded regions. The diameters of the fibers produced by electrospinning primarily depended on the spinning parameters, the most crucial being the solution concentration [34]. The smaller average diameter of the PCL/PdNP fibers can be attributed to an expected increase in conductivity, in agreement with previous works making use of metallic nanoparticles. Thus, in the case of nanoparticles of ZnO, the authors hypothesized that the solution was seen to have a larger charge capacity, and then to be driven by a stronger electric force along the fibers; therefore, smaller fiber diameters were obtained [35].

The SEM images of the films’ cross-sections, shown in Figure 1c,d, indicated the presence of compact structures that resulted from the annealing post-processing step, which was in good agreement with previous works [20,21,22]). 

**Figure 2:**
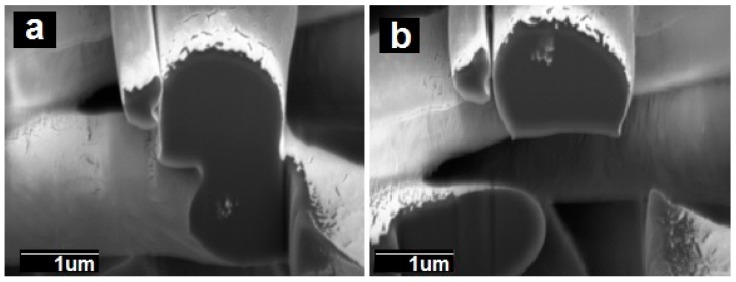
The cross-sectional SEM image of two PCL fibers with Pd nanoparticles after (FIB-SEM) sectioning. (**a**) PCL fiber 1 at the bottom with visible bright nanoparticles and (**b**) PCL fiber 2 at the top of the image with visible bright nanoparticles.

**Figure 3:**
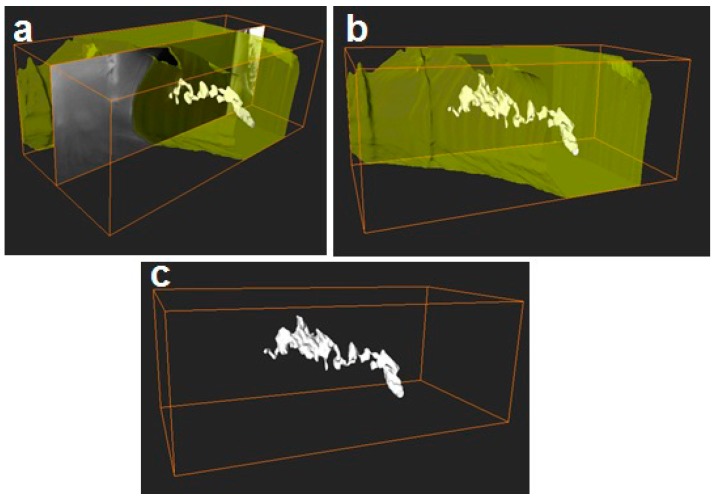
(**a**) The side view of 3D reconstructions of PCL fiber 1 (in semi-transparent yellow) and Pd nanoparticles (in white), including the SEM image (obtained from FIB sectioning) in the middle of the reconstruction, (**b**) 3D reconstruction of PCL fiber 1 with Pd nanoparticles inside, (**c**) 3D reconstruction of particles only inside of PCL fiber 1. The binding box size for this reconstruction had the following dimensions 3.105 × 1.185 × 1.526 μm.

Additionally, FIB-SEM was used to cross-section the internal structure of the electrospun fibers. The detailed examples of the FIB-SEM images of PCL /PdNP fibers collected during FIB sectioning are presented in Figure 2, showing that Pd particles are incorporated in the two fibers. The cross-sectional images enabled the visualization of the PCL/PdNP fibers in 3D, as shown in Figure 3. The Pd nanoparticles were seen to be distributed along the PCL fibers, forming small agglomerates at different parts within the fiber cross-sections. Finally, the concentration of particles was also estimated per the given volume of the piece of fiber analyzed, and for the PCL fiber 1, this was 1.1%, and for PCL fiber 2, this was 0.9%. These observations suggest that the PdNP are better dispersed and distributed across the PCL fibers, and that the agglomerates may account for most of the Pd in the nanocomposites.

In spite of the very revealing FIB-SEM results, the SEM technique is thought to be inadequate for resolving highly dispersed Pd nanoparticles within the polymer matrix [36]. In order to check for this, additional TEM experiments were conducted on the samples. 

**Figure 4:**
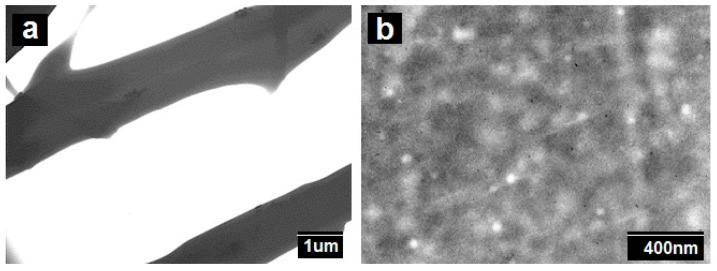
Scanning electron microscopy (TEM) images taken (**a**) directly on electrospun polycaprolactone (PCL) fibers containing palladium nanoparticles (PdNPs) and on (**b**) microtomed sections of their corresponding annealed film.

The additional TEM experiments displayed in Figure 4 indicate that the Pd nanoparticles, in agreement with the FIB-SEM experiments, exhibit a significant degree of aggregation within the fiber. Due to attractive forces (Van der Waals and others), particles tend to agglomerate, even in suspension, unless stabilized by equivalent repulsive forces such as surface charge or steric effects. Thus, the smaller the particle size, the greater the relative attractive forces per unit mass. This means that it becomes progressively more difficult to disperse nanoscale materials as the size decreases [37]. In any case, TEM also revealed the presence of some highly dispersed and distributed nanoparticles within the cross-section of the biopaper film. The smallest particles were seen to have diameters of ca. 6 ± 2 nm, and they seemed evenly distributed throughout the fibers/film. Our prior studies of PHB/PdNPs electrospun fibers, also showed a similar dispersion of Pd nanoparticles, with some clear agglomeration zones within the fibers [20].

### 3.2. FTIR Analysis of the PCL Electrospun Fibers and Films

**Figure 5:**
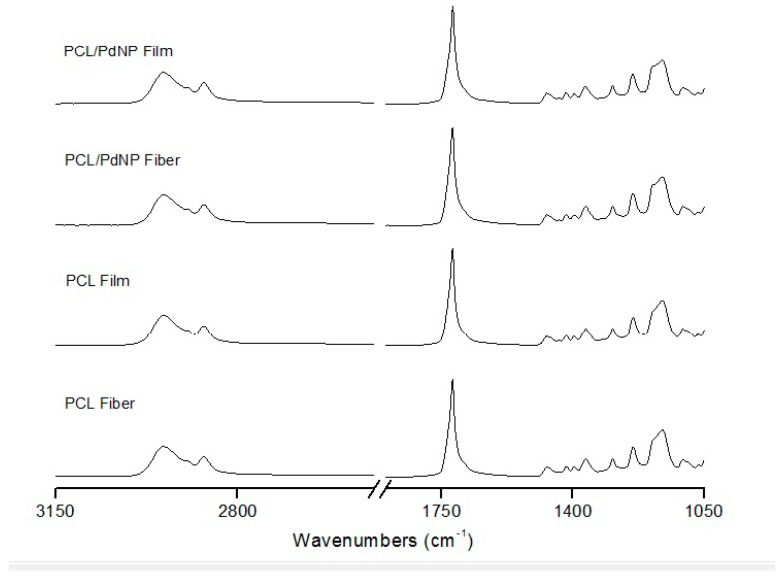
ATR-FTIR spectra of the electrospun PCL and the PCL/PdNP fiber mats and annealed films.

The FTIR spectra of the electrospun neat PCL fibers and film, and the PCL/PdNP fibers and film are shown in Figure 5.

The PCL spectrum displays the characteristic peaks of C=O stretching vibrations at 1726 cm^−1^, CH2 bending modes at 1361, 1397, and 1473 cm^−1^, and CH2 asymmetric stretching at 2942 and symmetric stretching at 2862 cm^−1^. The C-O-C stretching vibrations yield peaks at 1042, 1107 and 1233 cm^−1^. The bands at 1160 and 1290 cm^−1^ are assigned to C-O and C-C stretching in the amorphous and crystalline phases, respectively [38,39,40].

The overall PCL spectrum, including the main bands ascribed to PCL, such as the peaks at 2949 and 2865 cm^−1^ from methylene (CH2) groups, and the strong carbonyl (C=O) peak centered at 1720 cm^−1^, were not seen to be affected either by incorporating PdNP, nor by the post-processing step, suggesting a lack of changes across the polymer molecular backbone.

### 3.3. Thermal Properties of the PCL Electrospun Fibers

Table 1 shows the thermal properties, melting and crystallization points and enthalpies, in the first heating run and the subsequent crystallization run from the melt for the PCL and PCL/PdNP fibers mats. With the exception of the melting point, the rest of the thermal features were very similar for the neat PCL and nanocomposite fibers. The melting temperature for pure PCL is typically reported at 60 °C, and the glass transition temperature is −60 °C. As discussed, in the melting point, there was an increase of ca. 5 °C in comparison with the mean PCL, which must be explained by the addition of PdNPs to the polymer. This may due to the interaction of the polymer chains with the surface of the particles, which can change the chain kinetics in the region immediately surrounding the nanoparticles [41]. Similar results were reported by Bajsić et al. [42], where the melting point of the PCL/TiO_2_ composites was found to increase slightly with an increasing load of TiO_2_ micro- and nanoparticles.

**Figure 6:**
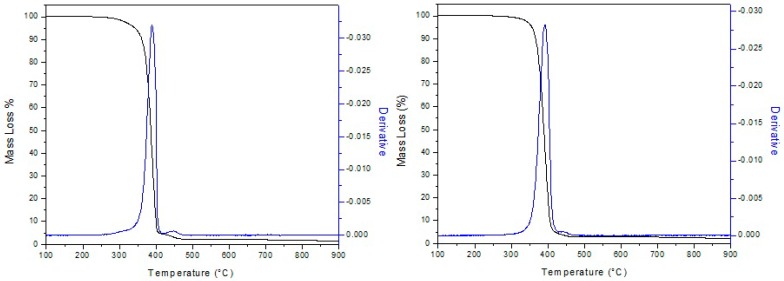
Thermogravimetric analysis (TGA) curves of the electrospun PCL (**left**) and PCL-containing palladium nanoparticles (PdNPs) fibers (**right**).

Thermogravimetric analysis (TGA) was carried out to evaluate the degradation temperature of the PCL and PCL/PdNP fibers, including the curves of the first derivative analysis (blue lines) (see Figure 6), and the results are summarized in Table 2.

From Figure 6, it can be observed that PCL and PCL/PdNP initiated degradation at 342 and 355 °C, respectively, exhibiting two transition peaks: The first transition peaks were at 388 and 391 °C, and the second transition peaks were at 449 and 447 °C, respectively. The residual material of PCL and PCL/PdNP had a slight difference of ca. 0.8%, which is ascribed to the Pd that is present in the sample.

The data in the work reported here indicate that adding 1 wt % PdNP resulted in a slightly higher degree of thermal stability for the composite. Previous studies showed that other metallic nanofillers can impact the degradation temperature of PCL in different ways. Thus, Wang et al. demonstrated that the thermal stability of PCL was depressed by the incorporation of Fe_3_O_4_/GO nanoparticles, most likely due to the filler acting as a catalyst for polymer degradation [43]. Castro-Mayorga et al. also observed that the degradation temperature of poly(3-hydroxybutyrate-co-3-hydroxyvalerate) (PHBVs containing ZnO nanoparticles showed lower degradation temperatures than that of pure PHBV3. This was attributed to the high thermal conductivity and catalytic properties of the ZnO nanoparticles [19]. Other studies suggest that a temperature drop can also be explained by the fact that nanoparticles can weaken to some extent the interactive force of polymer inter-chains, and hence assist the thermal decomposition of the nanocomposite [44]. However, in the current work, it was observed that the nanofiller induced a somewhat improved degree of thermal stabilization, which may be ascribed to a better adhesion between the nanoparticles and the polymer matrix, resulting in both a hindered diffusion of volatile decomposition products, and/or the sorption of these over the filler surface, for the nanocomposites. Thus, the effect of a filler in thermal stability is in fact thought to depend on the type, content, interfacial interaction, and the degree of dispersion and distribution of this into the polymer matrix [45]. Thus, Ag [45], Fe, and Zn nanoparticles [46,47] have also been previously reported to enhance the thermal stability of PHA, supporting the current results for PCL.

### 3.4. PHB Electrospun Fiber Morphology

**Figure 7:**
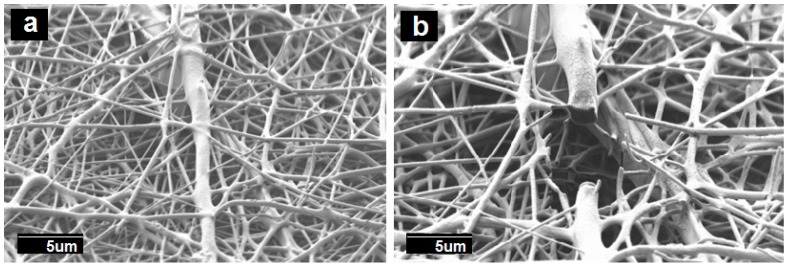
SEM images of the PHB sample. (**a**) Overview of PHB nanofibers, (**b**) PHB fibers after FIB sectioning.

Similarly to the FIB-SEM investigation of PCL fibers, the sectioning of PHB mats was also carried out, and it is displayed in Figure 7. The SEM images in Figure 7 indicate that the PHB fibers morphology is similar to the one reported in earlier work, with the fiber cross-section ranging between 200 and 400 nm, and showing a smooth and beads-free fiber morphology [20,21,22].

**Figure 8:**
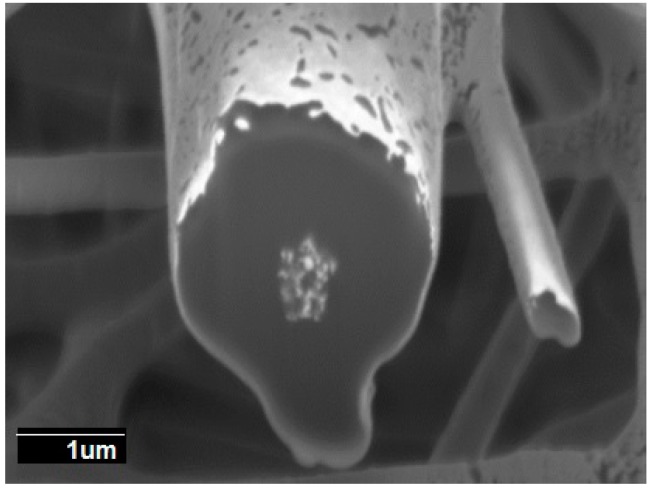
The cross-sectional SEM image of two PHB fibers with Pd nanoparticles after FIB sectioning.

**Figure 9:**
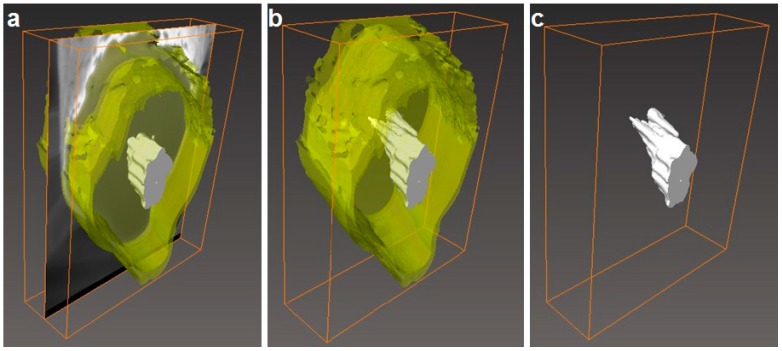
(**a**) The side view of the 3D reconstruction of the PHB fiber (in semi-transparent yellow) and Pd nanoparticles (in white), including the SEM image (obtained from FIB sectioning) in the middle of the reconstruction, (**b**) 3D reconstruction of the PHB fiber with Pd nanoparticles inside (**c**) 3D reconstruction of particles only from the inside of PHB fiber 2. The binding box size for this reconstruction had the following dimensions: 1.996 × 2.464 × 0.612 nm.

The cross-section of the individual PHB fiber shown in Figure 8 revealed that the PdNP agglomerated even more strongly than seen in PCL at the core of fiber, even with a surfactant, which was also visualized with the 3D reconstructions present in Figure 9. The 3D tomography allowed us to verify the presence of PdNP in the individual PHB fiber section scanned, similarly to the PCL data shown in Figure 3. The estimation of the Pd nanoparticle concentration of the fiber section analyzed yielded a concentration of 3%, suggesting that Pd is not as well-distributed as in PCL. This is a relevant finding that suggests that even when a surfactant was added to the PHB to improve filler distribution, as suggested by our earlier work [20], the distribution was still seen lower than for PCL without a surfactant. The reason for this could be explained by the different chemistry, but also by the fact that PHB is known to be a more rigid polymer that crystallizes to a greater extent into thick spherulites than PCL, which has more available free volume in the amorphous phase for dispersion. 

**Figure 10:**
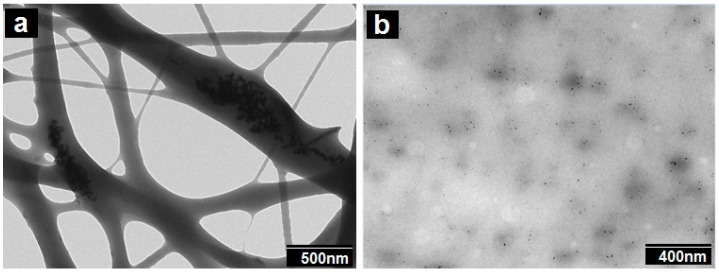
TEM images of (**a**) the PHB/PdNP fibers and (**b**) the PHB/PdNP film.

Figure 10 shows the TEM analysis of the fibers and the corresponding film, which are in agreement with the FIB-SEM results, in which some Pd agglomerations could be observed but also the presence of some Pd nanoparticles dispersed and distributed across the material matrix. These results are also in agreement with previous findings for PHB [20].

### 3.5. Morphology of the Multilayers

**Figure 11:**
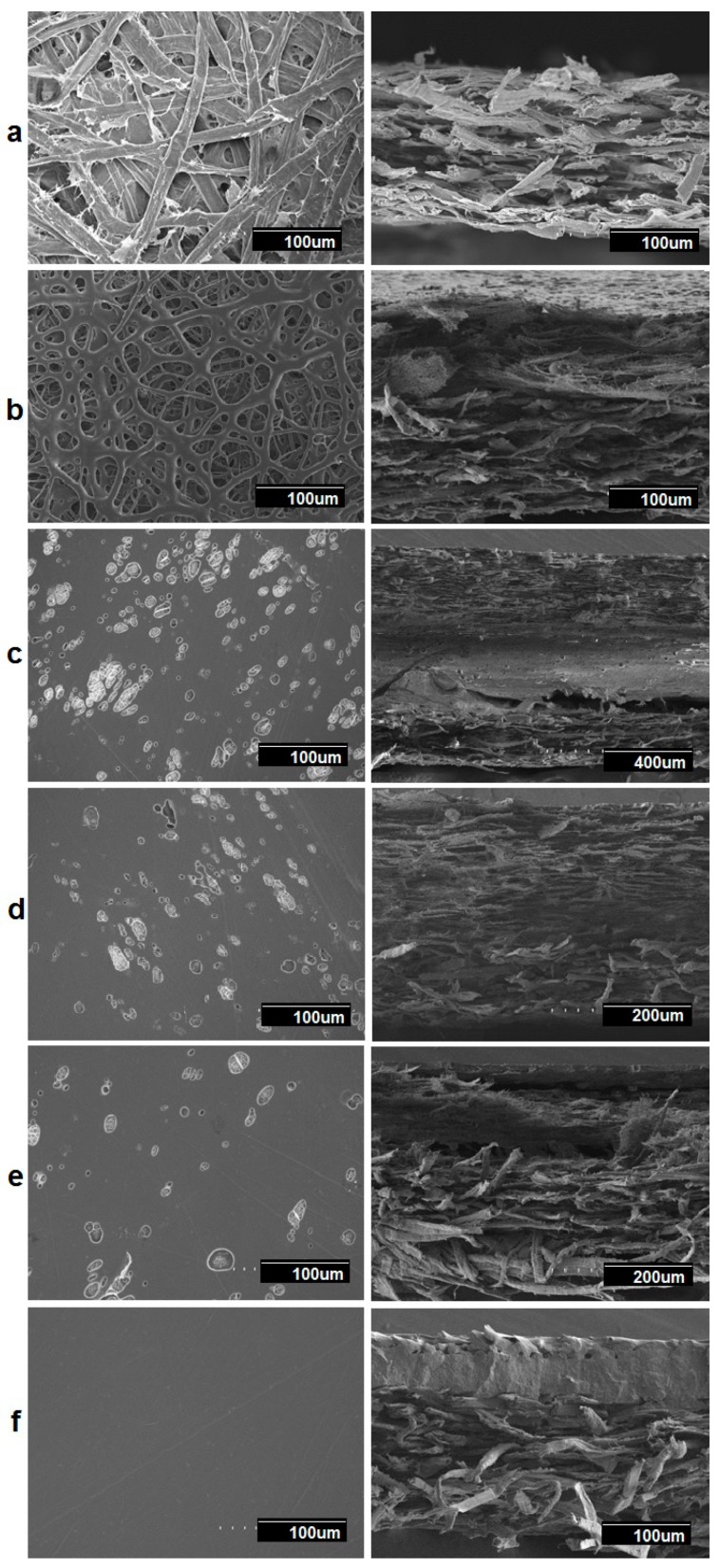
SEM images of the top view, and the cross section of (a) paper; (**b**) paper/PHB fibers/PCL fibers; (**c**) paper/PHB fibers/PCL-PdNP film; (**d**) Paper/PHB-PdNP fibers/PCL film; (**e**) Paper/PHB-PdNP fiber/PCL-PdNP film; (**f**) paper/PHB film/PCL-PdNP film.

Figure 11 shows the SEM images of the multilayer structures obtained, which contain a paper substrate, and electrospun PHB/PCL fibers containing PdNP and their annealed films. In all cases, the amount of PCL deposited (1 hr coating time) was lower than the amount of PHB (2 hr coating time) because PHB is a better water barrier than PCL. In a previous paper, PHB containing Pd nanoparticles showed a significant decrease in oxygen absorption after annealing; hence, in this work we intended to explore the feasibility of using some PCL in the coating, in the hope of enhancing the oxygen scavenging effect of Pd. As a result, the PCL layer has a lower thickness, and it was set as the top layer. After annealing, there is an additional packing of the fibers, resulting in an even lower layer thickness, as can be seen in Figure 11. 

The thickness of the paper layer was 117 μm and, of course, the fiber mean cross section of the paper was found to be higher, 18.37 ± 2.45 μm, compared to the electrospun fibers. Figure 11b shows that the surface of the electrospun neat PCL fibers exhibit significant fiber interconnections suggesting either remnant solvent induced coalescence and/or coarser fibers due to a drop in electric field as a result of deposition over other insulating materials as compared to direct deposition over the metallic collector (compare with Figure 1a). 

The multilayers presented in Figure 11c–f indicate the coalescence of fibers during the annealing step as expected, leading to a much less porous continuous film. Even though the samples had similar morphologies, it seems that sample (c) showed somewhat greater porosity, and sample (**f**) showed the least porosity. This was surely the effect of annealing in both coatings, leading to a higher level of packing structure.

In agreement with previous studies carried out on coatings with electrospun fibers over paper or polymer substrates, it is seen that the adhesion achieved after annealing between layers was very strong, due to the high surface-to-volume ratio of the electrospun fibers [22,48].

### 3.6. Passive and Active Barrier Properties of the Multilayers

#### 3.6.1. Water Vapor Passive Permeance

In general, the barrier properties of materials depend on the solubility and diffusion of the permeants, and hence, they depend on the permeant size, shape, and polarity; but also on the crystallinity, degree of cross-linking, and polymer chain segmental motion of the polymer matrix, among other factors [49,50].

The diffusion coefficient of water in an amorphous or semi-crystalline polymer is related to the particular molecular dynamics or segmental motions within the amorphous regions of the polymer. In addition, in semi-crystalline materials, a low crystallinity index and the formation of crystals of inferior quality confer a high degree of mobility to the macromolecular chains, resulting in lower barrier performance [51]. It is known that PHA is a better barrier material than PCL [52]. However, for fiber-based materials such as the paper/PHB fibers/PCL fibers multilayer generated here, the barrier performance was expected to be as low as paper, due to the existing porosity between the adjacent fibers. 

**Figure 12:**
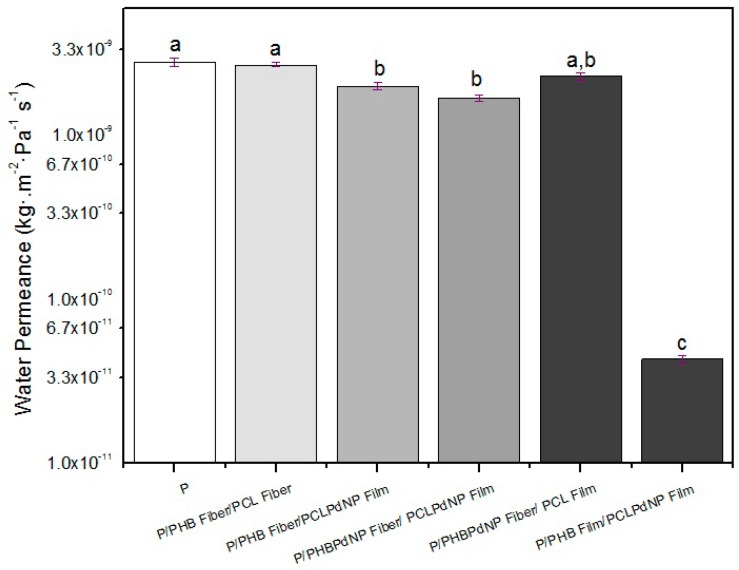
Water vapor permeance (WVP) of paper and Paper/PHB/PCL multilayers with and without palladium nanoparticles (PdNPs). Different letters indicate significant differences among samples (*p* < 0.05).

The water permeance data of the multilayer samples are gathered in Figure 12. From this figure, it can be clearly seen that the samples that contained fibers and that had porosity at the surface did not significantly enhance the barrier of the multilayers compared to neat paper. However, it can also be seen that when the two electrospun coatings underwent annealing, this led to a porosity reduction and stronger adhesion, and the water barrier performance was significantly increased.

The higher water barrier of the sample paper/PHB film/PCLPdNP film is then explained by a reduction in sample porosity, and also by the expected improvement in the crystalline morphology that occurs in the sample after annealing, which is known be both impermeable to the diffusion of sorbed water molecules, and impose restraints to the mobility of the amorphous phase [53]. The barrier data gathered for this sample (4.3 × 10–11 kg/m^2^·s^−1^·Pa^−1^) is in the same order of magnitude as the results obtained in a previous work for PHB (9.6 × 10–11 kg/m^2^·s^−1^·Pa^−1^) [20].

#### 3.6.2. Active Oxygen Scavenging Performance

**Figure 13:**
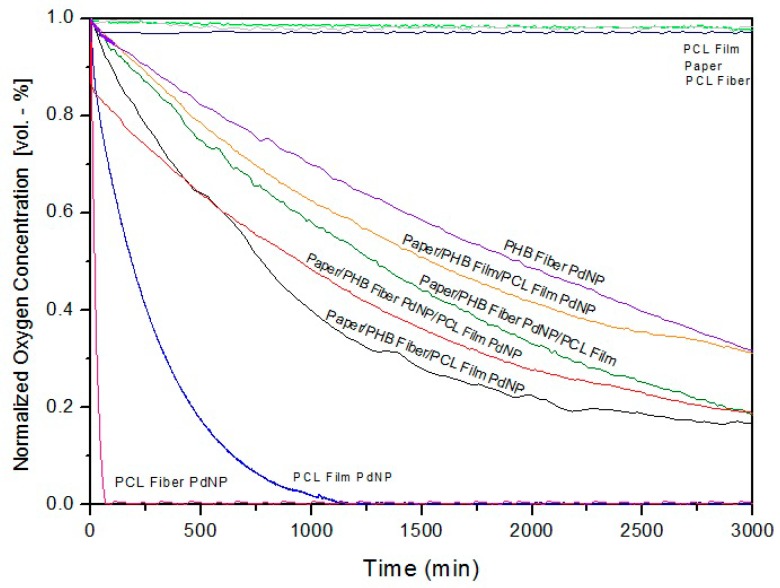
Oxygen depletion of PCL and PHB fibers, and paper/PHB/PCL multilayers and films with and without palladium nanoparticles (PdNPs). Values were measured at 50% relative humidity (RH).

Figure 13 shows the oxygen scavenging rate (OSR) of the PCL and PHB fibers, and the prepared paper based multilayers. The oxygen absorption of the fibers and resulting multilayers was investigated at 23 °C, with an initial oxygen concentration of 1.0% in the headspace of the measuring flasks, and at an RH of 50%. As reported earlier, due to the higher barrier of the material against water and oxygen compared to PCL, the PHB-PdNP fibers are not very efficient as oxygen scavengers at low or intermediate relative humidity, and even at high relative humidity as a film. However, PCL fibers and films are extremely quick at removing oxygen from the head space, suggesting that PCL is more adequate for hosting PdNP for oxygen scavenging purposes. The higher fractional free volume of the PCL polymer allows for moisture and oxygen to reach the catalyst quickly, and hence oxygen removal is more efficient. The greater reduction in the OSR of the fibers is, of course, related to the high surface-to-volume ratio of the electrospun fibers as compared to the annealed films. The dissociation rate of the hydrogen molecules into hydrogen atoms over the Pd surface in the films depends on the available surface area that is presented by the PdNP within the film.

As mentioned above, another important factor to be considered is that oxygen depletion is dependent on the RH conditions applied, and oxygen scavenging decreases when the RH increases [15,54]. In this work, we intended to conduct testing at medium relative humidities to simulate an intermedium case study. Figure 13 clearly indicates that, as expected, neither the paper nor the neat PCL polymer had any oxygen scavenging capacity.

Figure 13 also indicates that the best performing multilayer materials, in terms of OSR were, as expected, paper/PHB fibers/PCL-PdNP film and paper/PHB-PdNP fibers/PCL-PdNP film. In the cases where the intermediate layer is a PHB film or where the PdNP are not at the surface, the performance is reduced, since oxygen and moisture will possess slower kinetics of diffusion.

## 4. Conclusions

Electrospun nanobiopaper bilayer coatings of water barrier PCL and PHB biopolymers containing PdNP were here developed for the first time over conventional cellulose paper. Some of the materials were post-processed by annealing, to achieve both better adhesion among layers, and to form a continuous structure to improve the barrier properties to water. SEM and FIB-SEM results indicated that the PdNP agglomerated in certain areas of the fiber cross-section, but TEM results also indicated that some of the PdNP were dispersed and distributed within the biopolymer matrix. Better distribution of the PdNP was inferred for the PCL matrix. FIB-SEM 3D reconstruction was a very powerful tool for visualizing composites beyond the conventional SEM results, and it seen to exhibit good correspondence with TEM results. However, TEM was further able to resolve better at the nanoscale, and it showed that some of the PdNP were highly dispersed and distributed within the fibers. The water barrier was enhanced, as expected, after annealing of the fiber-based materials, but the annealing process also decreased the oxygen absorption capacity. A previous work indicated that with electrospun PHB fibers containing PdNP, the oxygen scavenging capacity of the films reduced to a significant extent after annealing, compared to the non-heated highly porous electrospun fibers. In this more advanced study on the topic, the oxygen scavenging of the PdNP was largely enhanced, even after annealing, by incorporating these within PCL, a more oxygen permeable material that is still biodegradable. 

The fully biodegradable fiber-based multilayered materials developed here show their tremendous potential for becoming the next generation of barrier papers, with demonstrated water barrier and oxygen scavenging capacity that are of interest in, for instance, food packaging applications. 

## Figures and Tables

**Table 1:** Thermal properties obtained by DSC in terms of melting temperature (T_m_), normalized melting enthalpy (ΔH_m_), crystallization temperature (T_c_), and crystallization enthalpies (ΔH_c_) for PCL and PCL/PdNP fibers.

Sample	T_m_ (°C)	ΔH_m_ (J/g)	T_c_ (J/g)	ΔH_c_ (J/g)
PCL Fibers	59.7 ± 1.2 ^a^	33.8 ± 2.0 ^b^	32.6 ± 0.9 ^b^	41.4 ± 2.1 ^b^
PCL/PdNP Fibers	64.7 ± 0.7 ^b^	27.8 ± 1.5 ^a^	31.2 ± 1.2 ^a^	38.2 ± 0.9 ^a^

**Table 2:** Values of thermal stability obtained from the thermogravimetric analysis (TGA) curves of the electrospun PCL and PCL/PdNP electrospun fibers in terms of degradation temperature at 5% of mass loss (T5%), maximum degradation temperature of the two degradation peaks (Td1, Td2), and residual mass at 900 °C (R900).

Sample	T5% (°C)	Td1 (°C)	Td2 (°C)	R900 (%)
PCL	342.5 ± 4.1	388.0 ± 5.3	449.3 ± 4.2	1.3 ± 0.05
PCL-PdNP	355.1 ± 4.5	391.7 ± 3.6	447.8 ± 5.3	2.1 ± 0.03
